# The BRCA1 Variant p.Ser36Tyr Abrogates BRCA1 Protein Function and Potentially Confers a Moderate Risk of Breast Cancer

**DOI:** 10.1371/journal.pone.0093400

**Published:** 2014-04-02

**Authors:** Charita M. Christou, Andreas Hadjisavvas, Maria Kyratzi, Christina Flouri, Ioanna Neophytou, Violetta Anastasiadou, Maria A. Loizidou, Kyriacos Kyriacou

**Affiliations:** 1 The Cyprus Institute of Neurology and Genetics, Department of Electron Microscopy/Molecular Pathology, Nicosia, Cyprus; 2 The University of Cyprus, Department of Biological Sciences, Nicosia, Cyprus; 3 The Cyprus Institute of Neurology and Genetics, Department of Clinical Genetics, Nicosia, Cyprus; CNR, Italy

## Abstract

The identification of variants of unknown clinical significance (VUS) in the *BRCA1* gene complicates genetic counselling and causes additional anxiety to carriers. *In silico* approaches currently used for VUS pathogenicity assessment are predictive and often produce conflicting data. Furthermore, functional assays are either domain or function specific, thus they do not examine the entire spectrum of BRCA1 functions and interpretation of individual assay results can be misleading. PolyPhen algorithm predicted that the BRCA1 p.Ser36Tyr VUS identified in the Cypriot population was damaging, whereas Align-GVGD predicted that it was possibly of no significance. In addition the BRCA1 p.Ser36Tyr variant was found to be associated with increased risk (OR = 3.47, 95% CI 1.13-10.67, P = 0.02) in a single case-control series of 1174 cases and 1109 controls. We describe a cellular system for examining the function of exogenous full-length BRCA1 and for classifying VUS. We achieved strong protein expression of full-length BRCA1 in transiently transfected HEK293T cells. The p.Ser36Tyr VUS exhibited low protein expression similar to the known pathogenic variant p.Cys61Gly. Co-precipitation analysis further demonstrated that it has a reduced ability to interact with BARD1. Further, co-precipitation analysis of nuclear and cytosolic extracts as well as immunofluorescence studies showed that a high proportion of the p.Ser36Tyr variant is withheld in the cytoplasm contrary to wild type protein. In addition the ability of p.Ser36Tyr to co-localize with conjugated ubiquitin foci in the nuclei of S-phase synchronized cells following genotoxic stress with hydroxyurea is impaired at more pronounced levels than that of the p.Cys61Gly pathogenic variant. The p.Ser36Tyr variant demonstrates abrogated function, and based on epidemiological, genetic, and clinical data we conclude that the p.Ser36Tyr variant is probably associated with a moderate breast cancer risk.

## Introduction

Breast cancer is the most frequent malignancy in the western world and even though most cases are sporadic, around 5–10% are believed to be hereditary caused by mutations in predisposing genes [1]. Germline mutations in the breast cancer susceptibility gene, *BRCA1* confer an estimated 60–85% and 40–60% lifetime risk of developing breast and ovarian cancer respectively by the age of 70 [2]–[5]. *BRCA1* is a tumor suppressor gene located on chromosome 17q21 [6] and encodes a multi-domain protein of 1863 amino acids which is involved in important cellular functions such as in DNA repair, transcription and cell cycle control through the DNA damage response [7]–[12].

Soon after the identification of the breast cancer predisposition genes *BRCA1* and *BRCA2*, mutation screening of families burdened with a substantial history of breast and ovarian cancer, became a routine demand, since it aids the identification of individuals at high risk. The outcome of such analysis may be a positive result (identification of a pathogenic mutation) or a negative result. However, in around 10% of cases an ambiguous result is obtained since variants of unknown significance (VUS), also known as unclassified variants, are identified [13], [14]. These are usually missense mutations whose effect on protein function is unknown. The identification of VUS causes problems in assessing cancer risk and complicates issues of genetic counseling, surveillance and targeted disease prevention in the affected carriers.

A number of approaches are being used to assess the clinical significance of VUS in the BRCA1 and BRCA2 genes. These include analysis of segregation of mutations with disease in families [15], evaluation of the frequency of variants in cases and unaffected controls [16], analysis of clinical and histopathological data [17], loss of heterozygosity analysis [18] and *in silico* predictions which are based on amino acid position and influence protein structure [19] as well as evolutionary conservation [20], [21]. Currently, classification of VUS in the *BRCA1* and *BRCA2* genes is based on integrated analyses employing multifactorial likelihood prediction models. These prediction models integrate several direct (frequency of variant in cases and controls, co-segregation of VUS with cancer in families, co-occurrence with a deleterious mutation in the same gene, personal and family history of cancer including age of onset and cancer type) and indirect (histopathology of associated breast tumors, loss of heterozygosity in tumor DNA, severity of the amino acid change and its conservation across species) factors in order to compute a posterior probability of pathogenicity [22]–[29]. However computational methods are predictive and therefore this kind of evaluation is not accurate as they do not actually examine the impact of a mutation on protein function *in vivo* or *in vitro*.

To overcome this problem many laboratories have established functional assays to assess the protein function of BRCA1 [30]. Morris and colleagues established a protocol for examining in parallel the interaction of the Really Interesting New Gene (RING) finger domain of a BRCA1 VUS with BARD1 and E2 UbCh5C in a yeast-two hybrid assay and its ability to promote *in vitro* formation of ubiquitin conjugates [31]. This assay relies on the production of BRCA1 and BARD1 recombinant protein domains in *E.coli* and the expression of fused BRCA1 and BARD1 RING domains in yeast and not of full-length protein sequences. Similarly, the yeast transcription activation assay is also based on the fusion of the BRCA1 C terminus (BRCT) domains only to the DNA binding domain of yeast GAL4 or of the viral repressor LexA and may not reflect the function of the whole BRCA1 protein sequence [9], [32]–[34]. For example two cancer associated mutations in the BRCT domain, the M1775R and Y1853X, have been shown to also affect the translocation of the protein into the nucleus [35], thus also affecting the nuclear shuttling of the protein controlled by the two nuclear localization signals (NLS) [36]. Likewise the phosphopeptide binding assay is also domain specific, as it only examines the ability of the BRCT domains to interact with small phosphorylated peptides [37], [38].

In contrast full-length BRCA1 protein sequence has been used in the radiation resistance assay [39], [40], in the homologous-directed recombination (HDR) assay [41] and in the embryonic stem cell based assay [42]. The human cell line HCC1937 which expresses truncated BRCA1 [43] exhibits hypersensitivity to ionizing radiation and upon introduction of wild type BRCA1 the hypersensitivity is abolished [40]. As many pathogenic BRCA1 variants exhibit ionizing radiation hypersensitivity, two groups have demonstrated that after co-culturing HCC1937 cells with MCF-7 transduced to co-express wild type or variant BRCA1 with a fluorescent protein following ionizing radiation, cells expressing functional BRCA1 outgrow HCC1937 cells [39], [40]. However this assay has not been evaluated yet. The HDR assay assesses the ability of a VUS to promote homologous recombination of double strand breaks (DSBs). Currently only VUS located in the RING domain have been screened but this assay needs further validation [41]. In addition, although the p.Arg71Gly variant was shown to be fully active in the HDR assay it is known to be pathogenic due to the production of an aberrant cryptic splicing site [44]. This highlights the fact that not a single assay can determine functional abrogation of a VUS, as different mechanisms may be affected by different variants irrespective of their location. The Sharan group developed elegant mouse models for examining VUS for both BRCA1 [42] and BRCA2 [45]. They engineered mouse embryonic stem cells with a knock-out allele and a conditional *BRCA1* allele. Lethality can be rescued by incorporation of the wild type *BRCA1* [42]. In this model variants that do not rescue lethality are considered pathogenic [42]. The limitations of this model are that lethality of human BRCA1 is examined in mouse and not in human embryonic stem cells; it is time consuming and if a variant is not lethal then functional assays, as well as multifactorial probability-based analysis still need to be performed for its classification.

The major drawback of many of the functional assays currently being used is that they are not interrogating BRCA1 function in a holistic manner, as they are restricted in expressing a specific domain or assessing a single specific function. These limitations are imposed by the large size of the full-length BRCA1 protein. Therefore multiple functional assays in combination with multifactorial probability-based analysis are usually necessary to decipher the pathogenicity and mechanism of action of a given variant. Nevertheless domain specific assays are useful for assessing the function of a given variant, especially when no other tools are available. In this manuscript, in an attempt to overcome these limitations, we describe a model of assessing the function of full-length BRCA1 protein in transiently transfected cells. This model can be adapted for screening multiple functions of BRCA1, overcoming the use of truncated forms of the protein which can lead to false observations. The proposed system enables the direct and simultaneous testing of the integrity of BRCA1 complexes, the sub-cellular localization of BRCA1 as well as the ability of BRCA1 to respond to genotoxic stress and migrate at sites of DSBs along with other interacting proteins of the DNA repair machinery. We hereby present the results obtained when this system was used to investigate the ability of p.Ser36Tyr BRCA1 novel VUS identified in 4 unrelated Cypriot families [46], to interact with BARD1, to promote the formation of ubiquitin chains following DNA damage and its sub-cellular localization.

## Materials and Methods

### Ethics Statement

The study was approved by the Cyprus National Bioethics Committee. Each participant gave written consent and the data were anonymized and coded into an MS ACCESS database.

### In silico predictions

*Ιn silico* analysis of the BRCA1 p.Ser36Tyr VUS was performed using:

Align-GVGD (http://agvgd.iarc.fr/agvgd_input.php) and

PolyPhen-2 (http://genetics.bwh.harvard.edu/pph2/index.shtml) algorithms.

### Analysis of BRCA1 p.Ser36Tyr VUS frequency in cases and unaffected controls

Unaffected females (n = 1174) aged between 40 and 70 years as well as sporadic breast cancer cases of the same age-range (n = 1109) ascertained by the MASTOS study [46] were screened for the presence of the BRCA1 p.Ser36Tyr missense mutation using a real time PCR based Taqman SNP genotyping assay. DNA samples from known carriers were used as positive controls. The study was approved by the Cyprus National Bioethics Committee. Each participant gave written consent and the data were anonymized and coded into an MS ACCESS database.

### Plasmid Construction

A detailed description of all the cloning procedures can be found in the Supporting Information S1.

### Cell culture

HEK293T cells were cultured in DMEM (Gibco, Life Technologies, Camarillo, CA, USA) supplemented with 10% FCS (First Link, UK), 2 mM L-Glutamine and the antibiotics penicillin (50 units/ml) and streptomycin (50 μg/ml) (Gibco, Life Technologies, Camarillo, CA, USA).

### Cell synchronization and introduction of DSBs with Hydroxyurea (HU)

For synchronization of cells in S-phase, cells were serum starved for 24 h in DMEM (Gibco, Life Technologies, CA, USA) and released for 24 h in DMEM containing 20% FCS (First Link, UK). Cells were treated with 3 mM HU (Sigma-Aldrich, Saint Louis, MO, USA) for 1 h at 37°C. The medium was removed and cells were allowed to recover in complete medium for 90 min before fixation or lysate preparation.

### Transient Transfection

HEK293T cells were transiently transfected using Lipofectamine 2000 or Lipofectamine-LTX (Invitrogen, Life Technologies, Camarillo, CA USA) according to the manufacturer's instructions. Successful transfection was verified by visualization of the dsRED fluorescent protein on a Carl Zeiss Inverted Fluorescent microscope (Carl Zeiss GmbH, Göttingen, Germany).

### Co-immunoprecipitation

For all co-precipitation experiments cells were grown in poly-L-lysine (Alamanda Polymers, Inc, Huntsville, US) coated tissue culture dishes (Nunc A/S, Thermo Fisher Scientific, Roskilde, Denmark). Cell monolayer was washed twice with PBS and cells were lysed on ice in 0.5% IGEPAL-CA 630 (Sigma-Aldrich, Saint Louis, MO, USA), 0.25 M NaCl, and 10 mM Tris/HCl (pH 7.5) in the presence of complete protease inhibitor cocktail (Roche Diagnostics GmbH, Mannheim, Germany). Where indicated, cytosolic and nuclear fractions were prepared using hypotonic buffer and cell extraction buffer (Invitrogen, Life Technologies, Camarillo, CA, USA) according to the manufacturer's instructions. The immunoprecipitation experiments were performed using 50 μl Dynabeads Protein G (Invitrogen, Life Technologies, Camarillo, CA, USA) per sample. In brief, the beads were washed in PBS-T (Tween-20 0.02%) and then incubated with rotation with 10 μg rat anti-DYKDDDDK Tag (FLAG) Antibody (Biolegend Inc, San Diego, US) at room temperature for 30 min. After antibody binding, beads were washed in PBS-T and incubated with rotation with the specified amount of lysate overnight at 4°C. Beads with immobilized antibody-protein complexes were washed 5 times in PBS-T and the final wash was performed with PBS alone. Protein complexes were eluted in 40 μl reducing Laemmli sample buffer and denatured at 95°C for 5 min.

### Western Blotting

Proteins were separated on 7.5% or 10% SDS/PAGE gels and electro-transferred on to nitrocellulose membranes which were blocked in 5% (w/v) non-fat dried skimmed milk. Membranes were incubated with either of the following primary antibodies: rat anti-DYKDDDDK Tag antibody (1∶1000, 3% BSA/PBS-T), rabbit anti-BARD1 (1∶1000, 3% BSA/PBS-T) (Santa Cruz Biotechnology, Heidelberg Germany), mouse anti-β actin (1∶10000, 5% milk/PBS-T) (Sigma-Aldrich, Saint Louis, MO, USA), mouse anti-GAPDH (1∶4000, 5% milk/PBS-T) (Santa Cruz Biotechnology, Inc., Heidelberg, Germany) or anti-lamin A/C (1∶200, 5% milk/PBS-T) (Novacastra, Leica Biosystems, Newcastle, UK). Membranes were washed 6 times for 5 min in PBS-T and then incubated with the appropriate HRP-conjugated anti- rat, mouse or rabbit antibody (1∶5000, 5% milk PBS-T) (Jackson Immunoresearch, Suffolk, UK). Membranes were washed as above and the chemiluminescence reaction was performed using either luminol (Santa Cruz Biotechnology, Inc, Heidelberg, Germany) or ECL-Plus (GE-Healthcare, Uppsala, Sweden) and exposed to photographic film or GelDoc imager (UVP Ltd, UK). Analysis method is described in the Supporting Information S1.

### Immunofluorescence Microscopy

For all immunofluorescence microscopy experiments cells were grown in 4-well Lab-Tek™ –CC2™ chamber slides (Nunc A/S, Thermo Fisher Scientific, Roskilde, Denmark). Cells were washed in PBS and fixed for 10 min at 4°C in 4% paraformaldehyde. Fixed cells were washed three times in PBS and permeabilized with 0.02% Tween/PBS for 5 min at room temperature. Cells were washed three times in PBS and blocked for 1 h in 20% FCS/PBS before incubation with antibodies. Antibodies were diluted in 20% FCS/PBS and cells were incubated with the primary antibody overnight at 4°C, washed three times in PBS and then 1 h at room temperature with the appropriate secondary antibody. Subsequently, cells were washed three times in PBS, stained with DAPI for 30 sec, washed twice in distilled water, cover slipped and examined on a Zeiss Axioplan-II microscope (Carl Zeiss GmbH, Göttingen, Germany) using a ×63 objective. The rat anti-DYKDDDDK Tag antibody was used to detect the exogenous expressed BRCA1 (used at 1∶500). The rabbit anti-BARD1 and Rad51 (Santa Cruz Biotechnology Inc, Dallas, US) were used at 1∶1000 and 1∶500 respectively. The FK2 mouse IgG antibody (Millipore, Upstate, Temecula, CA, USA) which recognizes only poly-ubiquitin chains was used at 1∶1000. The rat anti- DYKDDDDK Tag antibody (Biolegend Inc, San Diego, US) and the rabbit OctA (Santa Cruz Biotechnology Inc., Heidelberg, Germany), which also recognizes the FLAG sequence were used at 1∶500 and 1∶75 dilution respectively. Secondary antibodies used were the goat anti-rat IgG-AF647 (1∶50), goat anti-rabbit IgG-AF633 (1∶50) and donkey anti-mouse-AF488 (1∶1000), all from Invitrogen (Molecular Probes, Life Technologies, Camarillo, CA, USA) and the goat anti-rabbit IgG-AF488 (1∶500) by Jackson Immunoresearch (Suffolk, UK).

### Statistical analysis

A comparison of two proportions statistical analysis, comparing the test statistic to the normal distribution, was performed for each immunofluorescence experiment, comparing p.Ser36Tyr or p.Cys61Gly BRCA1 expressing cells with wild type BRCA1 expressing cells, in untreated/treated conditions with HU. At least 50 nuclei expressing exogenous BRCA1 were counted for nuclear co-localization with conjugated ubiquitin foci.

## Results

### *In silico* predictions and frequency of the BRCA1 p.Ser36Tyr variant in breast cancer cases and controls

Align-GVGD algorithm predicted that the *BRCA1* p.Ser36Tyr is a C15 class variant, with modest evidence in favor of pathogenicity. In contrast, according to the PolyPhen-2 algorithm, the *BRCA1* p.Ser36Tyr variant was classified as “probably damaging”.

The *BRCA1* p.Ser36Tyr variant was found 17 times in the MASTOS breast cancer case-control series [46], with an overall frequency of 1.2% in the breast cancer cases group (n = 13) and 0.34% (n = 4) in the unaffected controls. A statistically significant association between the p.Ser36Tyr variant and breast cancer risk was observed (OR = 3.47, 95% CI 1.13-10.67, P = 0.02).

Five female and a male breast cancer patient from 4 different families were heterozygous for the BRCA1 substitution p.Ser36Tyr. Four of five female patients, had a histological confirmed diagnosis of breast cancer at a relatively young age (33, 33, 44 and 51). The male breast cancer case was diagnosed at the age of 78. It is noted that the 4 pedigrees were small with only one or two affected individuals per family and the majority of the affected relatives were deceased at the time of study, hence it was difficult to assess co-segregation of the BRCA1 p.Ser36Tyr substitution with disease. It is also noteworthy that one out of the five affected female cases had a triple negative breast cancer which is associated with pathogenic *BRCA1* mutations.

### Expression of full-length BRCA1 and p.Ser36Tyr BRCA1 VUS in HEK293T cells

**Figure 1:**
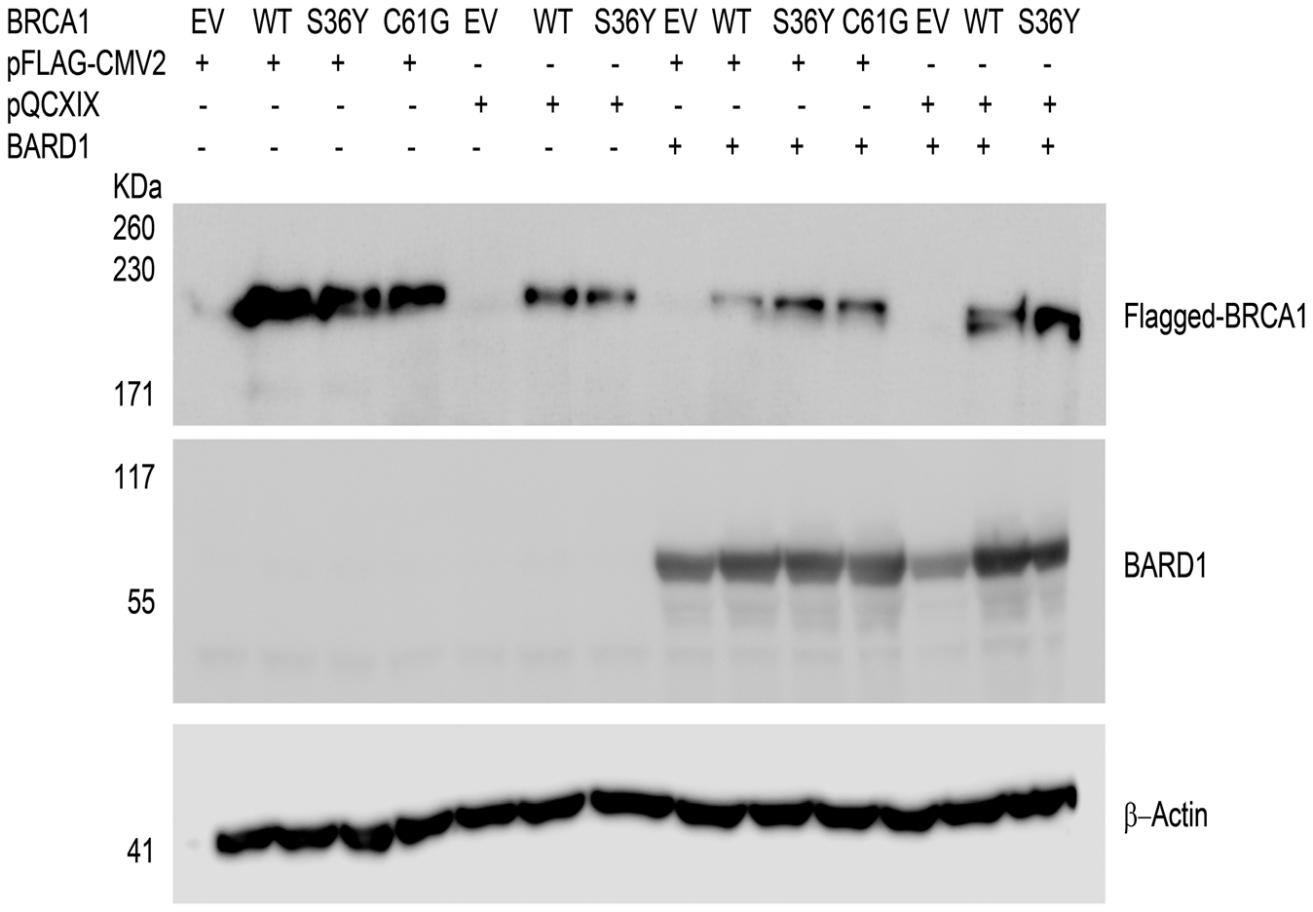
Expression of full-length, epitope-tagged BRCA1 in HEK293T cells. Protein expression analysis of wild type BRCA1, p.Ser36Tyr (S36Y) VUS and of the known pathogenic p.Cys61Gly (C61G) variant following transient transfection in HEK293T cells was confirmed with the anti-DYKDDDDK tag antibody that recognizes ectopically expressed BRCA1. Immunoblot analysis detected a 220 KDa band which corresponds to full-length BRCA1 protein. Both p.Ser36Tyr and p.Cys61Gly variants demonstrated reduced protein expression compared to wild type BRCA1. Co-transfection with wild type BRCA1 and BARD1 induced an increase in BARD1 protein expression (Supp Fig.1). EV corresponds to empty vector and WT to wild type protein. β-actin served as loading control. The results are representative of between 3 and 5 experiments.

A weakness in the accurate risk assessment of the clinical significance of *BRCA1* variants is the use of truncated protein sequences in functional assays instead of the full-length protein. For this purpose, the full-length cDNA of *BRCA1* was sub-cloned into the mammalian expression vectors pFLAG-CMV2 and pQCXIX with a FLAG sequence on the N-terminus. HEK293T cells were transiently transfected and full-length exogenous BRCA1 was readily detected in immunoblotting analysis (Fig. 1). The point mutation leading to the substitution of amino acid serine 36 into tyrosine did not appear to have an effect on the expression of full length protein similar to that of the p.Cys61Gly variant which is known to be pathogenic [47] (Fig. 1). Even though full-length BRCA1 p.Ser36Tyr was readily detected, the ectopic protein expression levels of this variant were not as high, as that of the wild type protein. The p.Cys61Gly variant also exhibited lower protein expression. It was further observed that BARD1 expression increased following co-transfection with wild type BRCA1 compared to empty vector, thus suggesting that co-expression of BRCA1 confers stability to BARD1 (Fig. 1 and Fig. S1).

### The interaction between the BRCA1 p.Ser36Tyr variant and BARD1 is compromised

**Figure 2:**
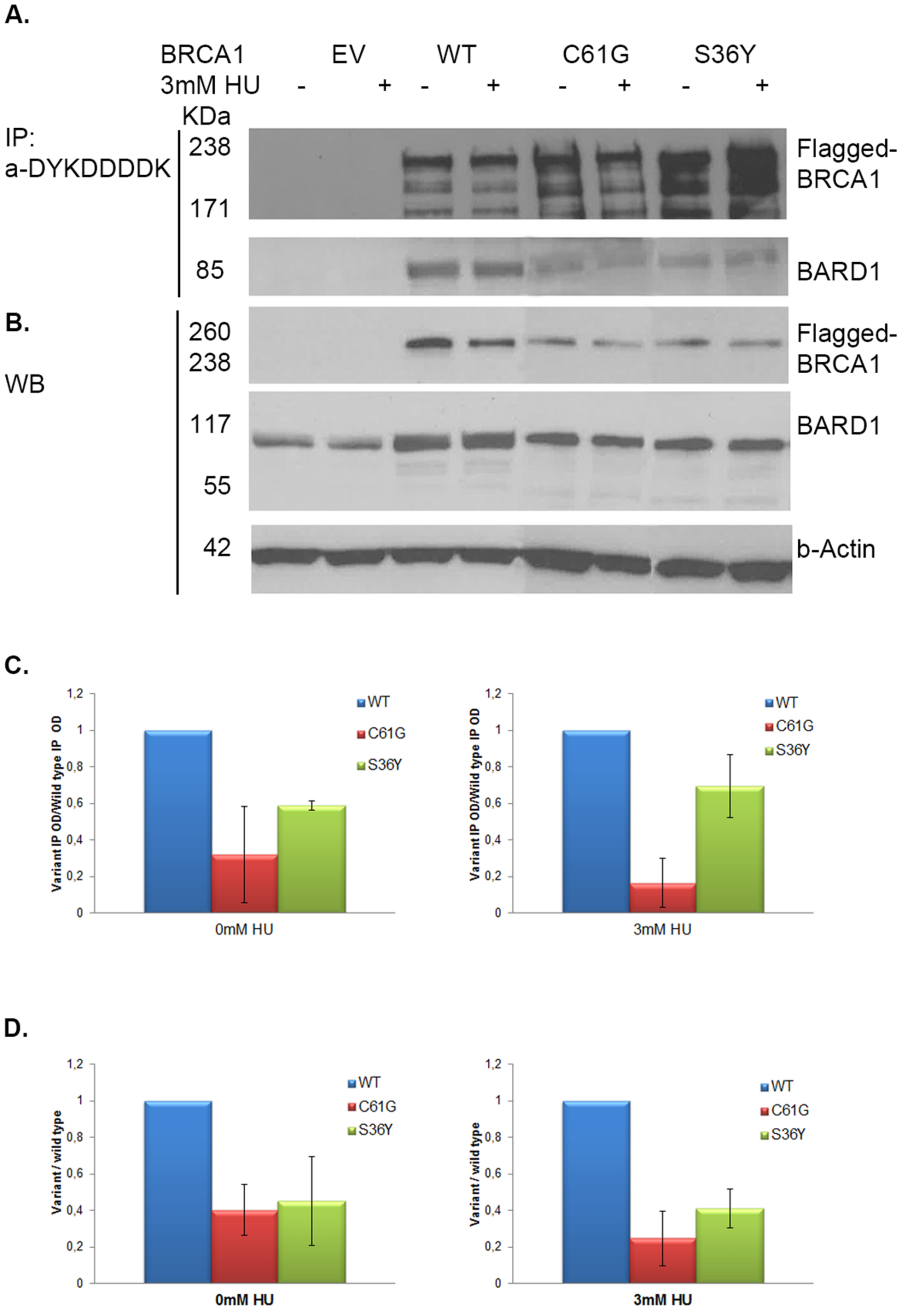
p.Ser36Tyr (S36Y) BRCA1 variant demonstrates reduced ability to interact with BARD1 in S-phase synchronized cells. A. Exogenously expressed BRCA1 co-precipitates with BARD1 in cells that were co-transfected with BRCA1 and BARD1, S-phase synchronized and treated with HU. Immunoblot analysis of the pull-downs performed with the anti-DYKDDDDK tag antibody which recognizes only the epitope-tagged exogenous BRCA1 protein, demonstrated that BARD1 levels co-precipitated by wild type BRCA1 are higher than those co-precipitated by each of the p.Ser36Tyr and p.Cys61Gly (C61G) variants in both resting or HU treated cells. B. Immunoblot analysis of whole cell extracts detected decreased BARD1 and exogenous BRCA1 expression in p.Ser36Tyr and p.Cys61Gly BRCA1 variant transfected cells compared to the wild type transfected cells. β-actin served as loading control. C. Bar charts comparing the levels of BARD1 co-precipitated by p.Ser36Tyr and p.Cys61Gly variant with wild type BRCA1 in untreated cells or cells treated with HU, demonstrate that both variants exhibit reduced ability to interact with BARD1 compared to wild type BRCA1. D. Bar charts showing the expression levels of wild type BRCA1 and p.Ser36Tyr and p.Cys61Gly variants following normalization with β-actin in whole cell extracts, demonstrate that both variants are expressed at lower levels compared to wild type BRCA1. The results are representative of 3 experiments.

BRCA1 has an intrinsic E3 ligase activity through the interaction of its RING domain with the respective RING domain of BARD1 [48]. The p.Ser36Tyr substitution falls within the RING domain of BRCA1 so the ability of this variant to interact with BARD1 was investigated. HEK293T cells were co-transfected with constructs encoding full-length BRCA1 and BARD1 and synchronized in the S-phase of the cell cycle. The BRCA1:BARD1 heterodimer was co-precipitated with the DYKDDDDK-tag antibody which recognizes the FLAG sequence on the N-terminus of BRCA1. As seen in Figs. 2A and 2C, the levels of BARD1 that co-precipitated with the Flag-tagged p.Ser36Tyr BRCA1 variant were almost two-fold lower than those that co-precipitated with wild type protein. The known pathogenic variant p.Cys61Gly also exhibited limited ability to interact with BRCA1 as expected (Figs. 2A and 2C). The reduced ability of the p.Ser36Tyr BRCA1 to co-precipitate and hence to interact with BARD1 was also observed in cells following treatment with HU, thus suggesting that during the process of DNA repair by homologous recombination the levels of the BRCA1:BARD1 complex are lower and the process may be impaired.

Western blotting analysis of the total content of exogenous BRCA1 protein in transfected cells demonstrated that the expression levels of the p.Ser36Tyr variant, as well as of the p.Cys61Gly pathogenic variant, were significantly lower than the levels of wild type protein (Figs. 2B and 2D), likely reflecting the reduced ability of these BRCA1 variants to precipitate similar amounts of BARD1 (Fig. 2C). These mutations caused decreased BRCA1 protein expression levels, and therefore protein availability during the DNA repair process is much lower.

**Figure 3:**
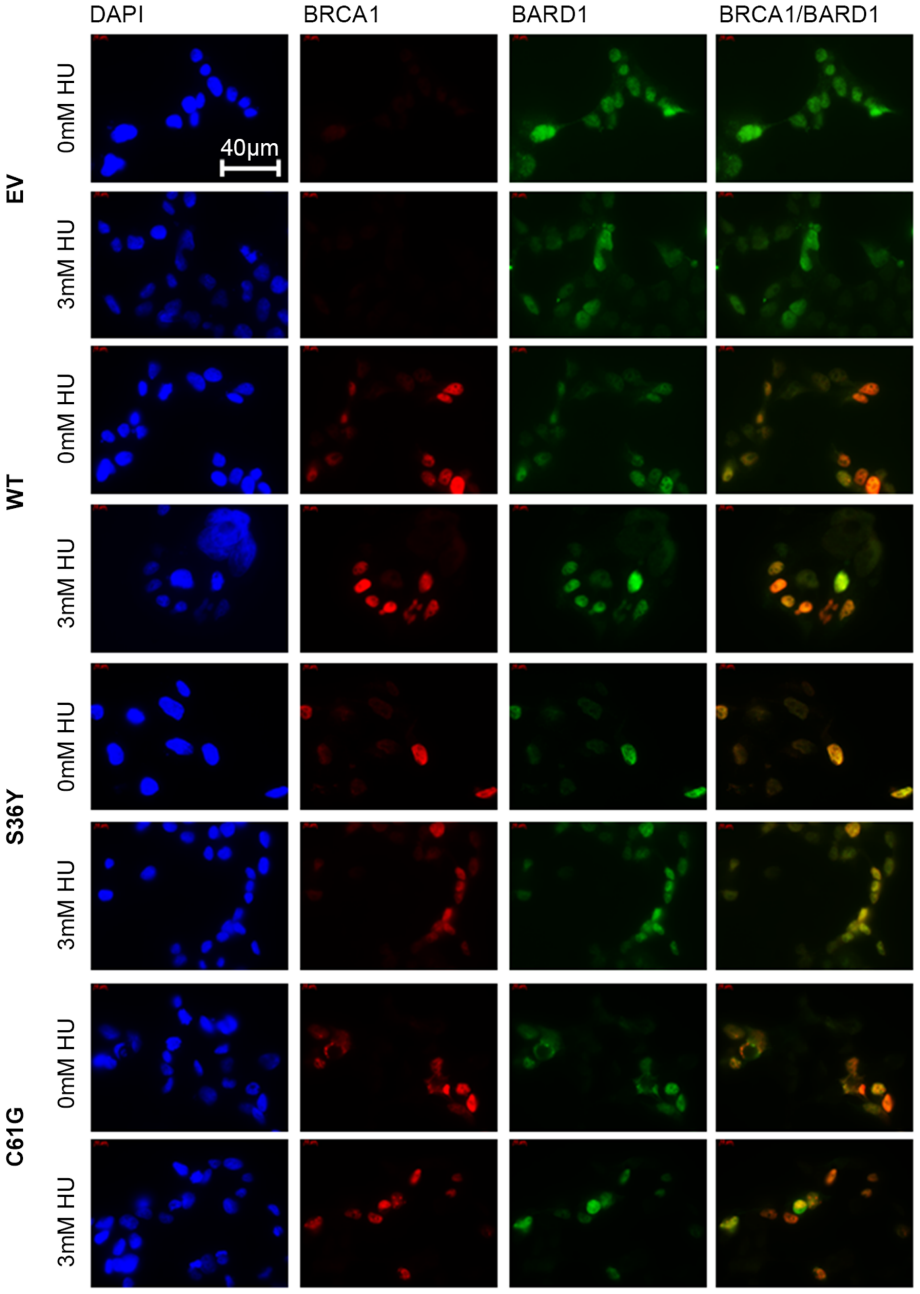
BRCA1 co-localizes with BARD1 in co-transfected HEK293T cells. Immunofluorescence analysis of transfected, S-phase synchronized cells following treatment with HU, demonstrated that the BRCA1 variants p.Ser36Tyr (S36Y) and p.Cys61Gly (C61G) exhibit lower expression levels in the nuclei of transfected cells in comparison to wild type (WT) transfected cells. However, neither of them lost the ability to co-localize with BARD1 in both HU untreated or treated cells. Nuclei were stained with DAPI (blue). When the pictures are merged, where green and red signal overlap a yellow signal is seen indicating co-localization. The results are representative of 3 experiments. Scale bar: 40 μm

In order to verify that the p.Ser36Tyr BRCA1 variant can form a complex with BARD1 in the nuclei of transfected cells, the localization of the two proteins following co-transfection and S-phase synchronization was examined by immunofluorescence microscopy. As seen in Fig. 3, the p.Ser36Tyr BRCA1 variant exhibited reduced expression levels in the nucleus of transfected cells compared to the wild type protein, but nevertheless it co-localized with BARD1, thus supporting the immunoprecipitation findings that the interaction is not entirely abolished. In HU-treated cells, the p.Ser36Tyr BRCA1 variant was not fully mobilized into the nucleus, and as a result exogenously expressed p.Ser36Tyr BRCA1 was also present in the cytoplasm (Fig. 3). The known pathogenic variant p.Cys61Gly also exhibited lower protein expression in the nucleus, but still co-localized with BARD1. However, in many cells BARD1 foci were also independent of BRCA1 p.Cys61Gly foci (Fig. 3).

**Figure 4:**
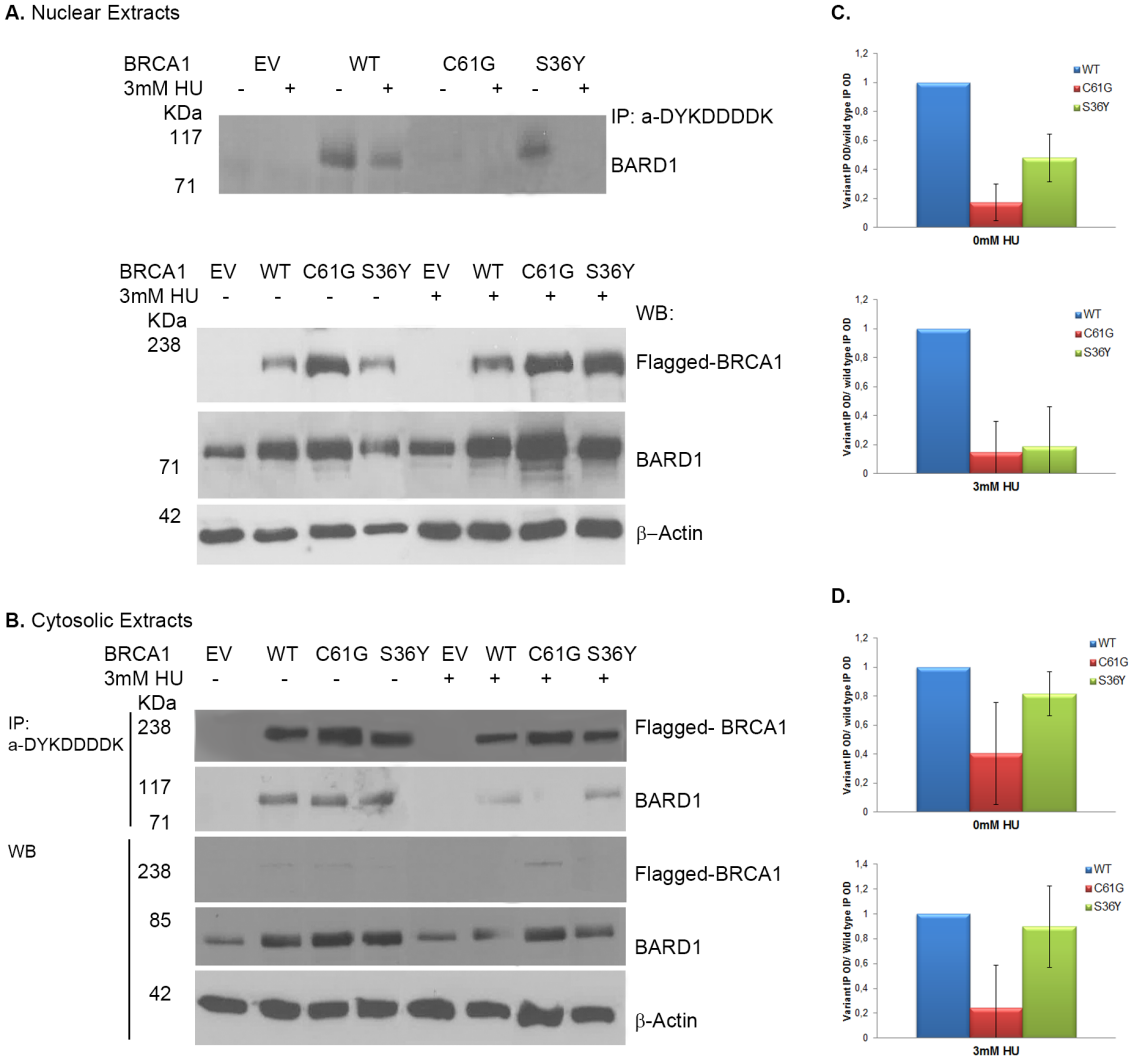
p.Ser36Tyr (S36Y) BRCA1:BARD1 complex is withheld within the cytoplasm following HU treatment. Lysates of transfected cells were fractionated into nuclear and cytosolic extracts following S-phase synchronization and treatment with HU. BARD1 was co-precipitated with the ectopically expressed BRCA1 in the A. Nuclear and B. Cytosolic extracts with the anti-DYKDDDDK tag antibody which recognizes only the exogenous BRCA1. A. Immunoblot analysis of the pull-downs demonstrated that unlike wild type BRCA1, both the p.Ser36Tyr and p.Cys61Gly (C61G) variants exhibit reduced ability to co-precipitate BARD1 in the nuclear extracts of stressed cells. B. However, in the cytosolic extracts the p.Ser36Tyr variant, but not p.Cys61Gly, co-precipitated a higher proportion of BARD1 in treated cells compared to wild type transfected cells. In untreated cells, both variants, including wild type BRCA1, co-precipitated larger amounts of BARD1 compared to treated cells. Bar charts showing that C. in the nuclei of treated cells, both the p.Ser36Tyr and p.Cys61Gly variants exhibit reduced ability to co-precipitate with BARD1, whereas D. in the cytosol the p.Ser36Tyr variant co-precipitates similar levels of BARD1 compared to wild type BRCA1, but it is not statistically significant. Immunoblot analysis of nuclear and cytoplasmic extracts demonstrating their purity is shown in Fig. S2. The results are representative of 2 experiments.

As the DNA repair process takes place within the nucleus of damaged cells, the ability of the p.Ser36Tyr BRCA1 variant to co-precipitate BARD1 in the nuclear extracts of damaged cells as well as the presence of the complex in the cytosolic extracts was investigated. Surprisingly, the levels of co-precipitated BARD1 by the p.Ser36Tyr variant in the cytosolic extracts of DNA damaged cells was higher than those that co-precipitated with wild type BRCA1 (Figs. 4B and 4D), whereas in the nuclear extracts the amount of BARD1 that co-precipitated with the p.Ser36Tyr variant was very low or barely detectable (Figs. 4A and 4C). It should be noted though that statistical analysis of two independent experiments, demonstrated that the increased BARD1 levels co-precipitated by p.Ser36Tyr in the cytosol are not significant. The finding that low levels of BARD1 co-precipitated with the p.Ser36Tyr variant in the nucleus of HU-treated cells, suggests that the presence of either of the two or both proteins is a limiting factor. However Western blotting revealed that both proteins were present in the nucleus (Figs. 4A and 4C). This suggests that the physical interaction of the p.Ser36Tyr BRCA1 variant and BARD1 in the nucleus is compromised by this substitution.

Immunofluorescence analysis of transfected S-phase synchronized cells further supported the above observations. In many cells ectopically expressing the p.Ser36Tyr BRCA1 variant, both BRCA1 and BARD1 were not entirely mobilized to the nucleus, as both proteins were also present in the cytoplasm (Fig. 3). The retention of BRCA1 p.Ser36Tyr variant in the cytoplasm of transfected cells was more pronounced following genotoxic stress with HU in S-phase synchronized cells, thus explaining the failure of this variant to co-precipitate high levels of BARD1 in the nuclear extracts of damaged cells.

These observations suggest that the mutation causing the amino acid change at position 36 from serine to tyrosine not only reduces BRCA1 protein expression, but it also causes partial retention of the protein within the cytosol and a high proportion of the BRCA1:BARD1 heterodimer fails to migrate to the nucleus of damaged cells.

### Co-localization of p.Ser36Tyr BRCA1 nuclear foci with conjugated ubiquitin foci in stressed cells is severely impaired

**Figure 5:**
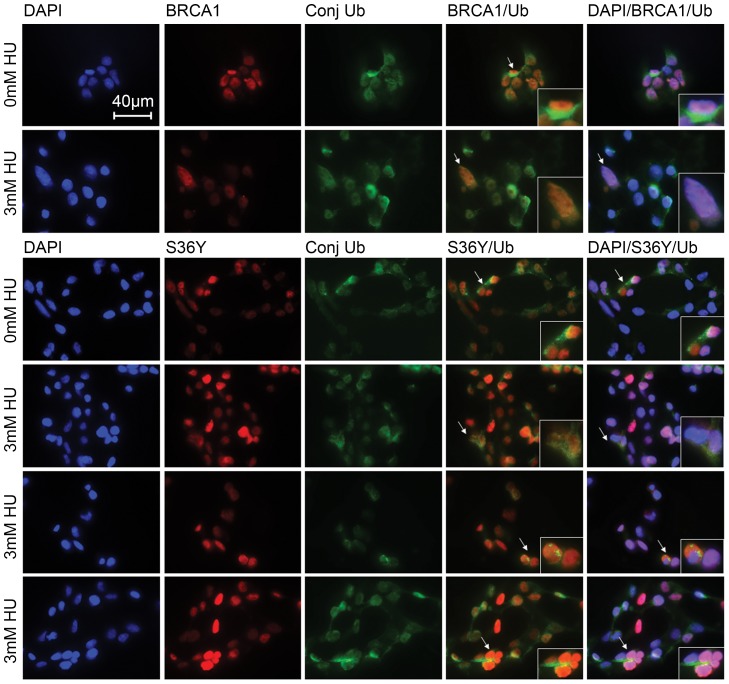
p.Ser36Tyr (S36Y) BRCA1 variant exhibits diminished co-localization with conjugated ubiquitin foci in HU treated cells. Immunofluorescence analysis of transfected, S-phase synchronized cells demonstrated that the p.Ser36Tyr BRCA1 variant fails to co-localize with conjugated ubiquitin detected by the FK2 antibody that recognizes only conjugated ubiquitin structures. A high proportion of p.Ser36Tyr BRCA1 and conjugated ubiquitin were detected outside the nucleus similar to wild type transfected cells. HU treatment induced mobilization and co-localization of wild type BRCA1 and conjugated ubiquitin in the nuclei of transfected cells. Contrary to the wild type transfected cells, HU treatment did not induce p.Ser36Tyr nuclear mobilization to the same levels and in many cells the p.Ser36Tyr protein did not co-localize with conjugated ubiquitin foci. Three different locations are shown for the p.Ser36Tyr variant following treatment with HU. Nuclei were stained with DAPI. The forth column is the merge of BRCA1 and conjugated ubiquitin and where green and red signals overlap a yellow signal is seen indicating co-localization. The fifth column is the merge of all stains. Where all signals overlap a white signal is seen, where red and blue signal overlap a pink signal is seen and where green and blue signals overlap a violet signal is seen indicating co-localization. p.Cys61Gly (C61G) and empty vector (EV) control are displayed in Supporting Fig. 3). Statistical analysis confirmed that the observed effects are significant (p<0.05). Insets show the arrow pointed cells following enlargement. The results are representative of 3 experiments. Scale bar: 40 μm.

The E3 ligase activity of the BRCA1:BARD1 heterodimer is enhanced during the S-phase of the cell cycle after genotoxic stress [49]. During this process, Morris and colleagues demonstrated that the levels of conjugated ubiquitin structures that co-localize with BRCA1 in the nucleus increase and BRCA1 or BARD1 knockdown inhibits their formation in irradiated or HU-treated cells [49]. In light of the above observations, the p.Ser36Tyr BRCA1 can still interact with BARD1 but not as efficiently as wild type protein. However, it is not known whether this variant maintains the E3 ligase activity of the heterodimer and induces the formation of conjugated ubiquitin chains at sites of DNA damage. The ability of the p.Ser36Tyr BRCA1 variant to promote the formation of conjugated ubiquitin foci in S-phase synchronized cells was investigated. As expected, wild type BRCA1 co-localized with conjugated ubiquitin foci in resting cells and the levels of conjugated ubiquitin foci increased following treatment with HU (Fig. 5). In contrast, cells ectopically expressing the p.Ser36Tyr BRCA1 variant exhibited a reduced ability to form conjugated ubiquitin foci in the nucleus. These effects were further enhanced following treatment with HU, as in treated cells the foci of conjugated ubiquitin chains barely co-localized with ectopically expressed BRCA1 p.Ser36Tyr, and in many cells it was noticed that conjugated ubiquitin chains formed outside the nucleus or were completely absent (Fig. 5). The impaired formation of conjugated ubiquitin foci in p.Ser36Tyr expressing cells is more evident than that caused by the pathogenic p.Cys61Gly variant (Fig. S3), and a comparison of two proportions statistical test confirmed that the effect is statistically significant (p<0.05) in three independent experiments.

The p.Ser36Tyr BRCA1 variant impairs the formation of conjugated ubiquitin foci in the nucleus of S-phase synchronized cells following genotoxic stress hence has an effect on the E3 ligase activity of the BRCA1:BARD1 heterodimer.

## Discussion

Genetic screening for mutations in the breast cancer susceptibility genes *BRCA1* and *BRCA2* for families at high risk is a well established diagnostic modality. Nevertheless, such screening does not always produce a conclusive answer, as in about 10% of individuals opting for the test a VUS is identified [13], [14]. The identification of a VUS in the *BRCA* genes is a major issue for geneticists, counsellors, oncologists and carriers. In contrast to the families carrying deleterious mutations, where relatives can be offered predictive testing, and carriers can benefit from risk-reducing interventions and/or targeted surveillance, such management is not possible in families with a VUS, and surveillance can only be based upon the extent of the cancer family history. Therefore, it is of utmost importance to assess the clinical significance of each variant. Currently the pathogenicity of a VUS is widely assessed by multifactorial probability-based model analysis (reviewed by [50]), and ideally in combination with functional assay data. However these tools are predictive and the *in vivo* behavior of the protein is not scrutinized. Although functional assays have been established, most are domain- and function- specific. Indeed, because BRCA1 is a multi-domain protein involved in a number of key cellular processes, the results of such assays can often be misleading and may even result in inaccurate cancer risk assessment, as they do not take into account the possible impact on the function of the entire protein. In order to overcome these limitations we have set up an *in vitro* cellular system where we can achieve high protein expression levels of full-length BRCA1 following transient transfection. This approach can form the platform for setting up a wide range of functional assays which will enable the simultaneous interrogation of the known key functions of the intact BRCA1 protein.

Bioinformatics analysis, using PolyPhen and Align-GVGD yielded contradictory predictions regarding the pathogenicity of the p.Ser36Tyr BRCA1 variant, identified in 4 Cypriot families and in 13 cases of sporadic breast cancer [46]. This gave us the motivation to set up a cellular system for classifying this variant. In our system, we have successfully cloned the full-length coding sequence of BRCA1 into a mammalian expression vector with an epitope sequence on its N-terminus to allow detection of exogenously expressed protein. We demonstrated high protein expression levels of the full length BRCA1 in transiently transfected HEK23T cells. Similarly, we detected full-length protein for the p.Ser36Tyr variant and the known pathogenic variant p.Cys61Gly. However, the expression levels of both variants were lower, compared to the wild type protein. This suggests that these mutations may either interfere with protein expression or that the resulting proteins are not as stable as wild type BRCA1. Given these observations then it is likely that the p.Ser36Tyr variant is clinically significant due to protein instability.

**Figure 6:**
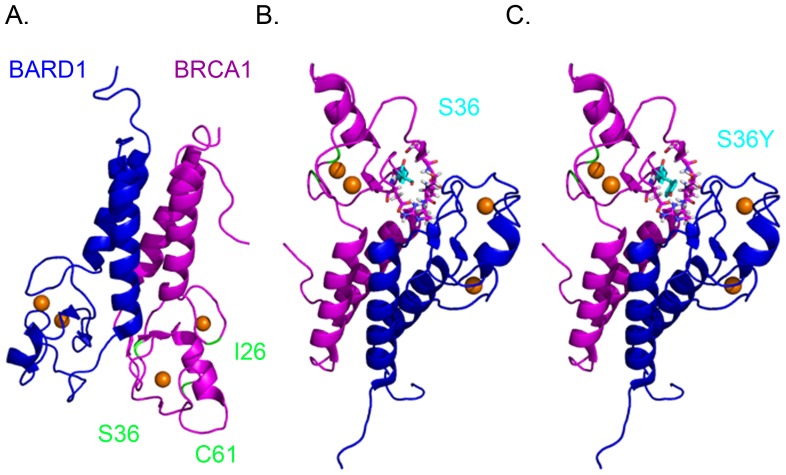
Ribbon representation of the BARD1:BRCA1 complex. A. The RING domains of BRCA1 and BARD1 (PDB 1JM7 [63]) are represented in magenta and blue respectively and the residues isoleucine 26, serine 36 and C61G are indicated in yellow. Orange spheres represent the Zn^2+^ ions. This model demonstrates that serine 36 lies within the second β-sheet of the RING structure. B. and C. represent the complex in a different orientation revealing the position of serine 36 (B.) and its substitution by tyrosine (C.) may distort the structure. The model was modified using PyMOL software.

The heterodimerization of BRCA1 with BARD1 is believed to confer stability to BRCA1 and their interaction is mediated through their RING domains [12], [51]. As the p.Ser36Tyr mutation falls within the RING domain of BRCA1 we investigated whether the mutation affects the interaction with BARD1. Co-precipitation analysis demonstrated that the interaction is not abolished, but the levels of co-precipitated BARD1 in S-phase synchronized cultured cells, were not as high as those precipitated by the wild type protein. This was expected as p.Ser36Tyr BRCA1 exhibited lower protein expression compared to the wild type. Nevertheless it is not known if the affinity of the p.Ser36Tyr variant for BARD1 was decreased. The findings were further supported by the immunofluorescence data as BRCA1 nuclear foci co-localized with BARD1 foci, in S-phase synchronized cells and were mobilized into more concentrated foci after treatment with HU. Despite the nuclear localization of BRCA1 in S-phase synchronized cells, it was observed that in many cells BRCA1 was also located in the cytoplasm and this was more evident in the p.Ser36Tyr variant transfected cells. Cell fractionation into nuclear and cytosolic extracts, revealed that a proportion of the p.Ser36Tyr BRCA1:BARD1 complex remained within the cytoplasm and its cytoplasmic retention was more pronounced following genotoxic stress with HU. Within the RING domain of BRCA1 there are two nuclear export sequences (NES) that signal BRCA1 shuttling to the cytoplasm [52], [53] and downstream the RING domain there are two nuclear localization sequences (NLS) [36]. BRCA1 shuttling in and out of the nucleus is cell-cycle dependent; and its import to the nucleus can take place by two independent mechanisms: either through the importins-α/β which are dependent on GTP hydrolysis and binding of importin-α to the NLS (501-508) of BRCA1 [36], or through its interaction with BARD1, where the NLS of BARD1 mediates the translocation [54]. Its nuclear export is mediated by the interaction of the NES of BRCA1 with the CRMI nuclear export receptor [52] and this process is inhibited when BRCA1 is in complex with BARD1 [54]. It is plausible that the reduced ability of the p.Ser36Tyr variant to translocate into the nucleus might be due to an interference with one of the nuclear shuttling mechanisms. This mutation may cause conformational changes to the protein that mask the NLS binding site for importin-α, or the transport mechanism with BARD1 may be impaired, due to protein instability or weaker protein-protein interaction due to lower affinity, or conformational changes. The NES of BRCA1 is masked through its interaction with BARD1, thus blocking the export of BRCA1 from the nucleus [54]. It is plausible that the p.Ser36Tyr change induces conformational changes that result in partial blocking of the NES, thus directing the heterodimer into the cytoplasm. Serine at position 36 is located in the β-sheet of the RING domain (Fig. 6) and it is likely that its substitution by tyrosine disrupts the structure and causes an overall conformational change.

The heterodimer BRCA1:BARD1 exhibits E3 ligase activity through the RING domain of BRCA1 as it promotes the formation of polyubiquitin conjugates in collaboration with the UbcH5C E2 enzyme [48], [55], [56]. Following genotoxic stress, BRCA1's ligase activity is further enhanced by SUMO modification [57]. The E3 ligase activity of the heterodimer has been implicated in DNA damage repair by homologous recombination during the S-phase of the cell cycle. Indeed it has been shown that foci of BRCA1, conjugated ubiquitin and γH2AX co-localize in the nucleus of S-phase synchronized cells, following genotoxic stress [49]. The p.Ser36Tyr BRCA1 variant also demonstrated a reduced ability to promote formation of conjugated ubiquitin foci at sites of DNA damage. Compared to wild type BRCA1, this variant failed in most cases to co-localize with conjugated ubiquitin chains, and this failure was even more pronounced than that of the known pathogenic variant p.Cys61Gly, which appears to have some residual activity as it can co-localize with BARD1, conjugated ubiquitin foci and Rad51 (data not shown), in agreement with the findings of Drost and colleagues [58]. Our findings prompt us to suggest that the p.Ser36Tyr variant exhibits impaired E3 ligase activity. However, at present it is not known whether this is due to protein instability, inability to interact with the E2 enzyme, impaired shuttling into the nucleus, or low levels of BRCA1:BARD1 complex. It is even possible that the substitution of serine by tyrosine generates a target for another kinase(s) that alters phosphorylation events. The known pathogenic p.Ile26Ala variant, is located within the binding interface with E2 enzyme and this substitution abolishes this interaction E2 [59], whereas p.Cys61Gly disrupts the interaction with BARD1 [47]. We believe that the substitution of serine 36 by tyrosine disrupts the β-helix of the RING domain of BRCA1 causing an overall conformational change that affects both interactions with BARD1 as well as with E2 enzyme. The functional data in this work suggest that the abrogated protein function exhibited by the p.Ser36Tyr BRCA1 variant is due to a combination of effects on the interaction with BARD1 and E2.

The E3 ligase activity of the BRCA1:BARD1 heterodimer is believed to be implicated in tumor suppression, as missense mutations in the RING domain of BRCA1 that disrupt the interaction with E2 have been reported in patients [31]. However, it was demonstrated in a mouse model that mutations in the BRCT domain and not E3 ligase inactivating mutations cause a high rate of tumor formation [60]. This observation prompted the investigators to suggest that mutations in the RING domain that disrupt the E3 ligase activity of BRCA1 are not linked to breast cancer predisposition, whereas those that disrupt the interaction with BARD1 are linked to tumor development [60]. Therefore it is still unclear which of the known functions of BRCA1 are directly involved in tumor suppression and this further complicates the accurate assessment of cancer risk for BRCA1 VUS. In consideration of the findings of the present study in combination with the classification of other variants that exhibit similar behavior to that of the p.Ser36Tyr (i.e. p.Ile26Ala E3 ligase inactivating mutation due to loss of interaction with E2 enzyme [59] and p.Cys61Gly due to impaired interaction with BARD1 [47]), our experimental evidence supports that the p.Ser36Tyr substitution abrogates the function of BRCA1 protein.

The superiority of functional assays over bioinformatics tools for classifying a VUS, is highlighted in this work, as discrepancies were exhibited using two of the most widely applied software analysis tools PolyPhen and Align-GVGD, to classify the p.Ser36Tyr variant. PolyPhen predicted that both p.Ser36Tyr and p.Cys61Gly are possibly damaging [61], whereas Align-GVGD predicted that the probability of the p.Ser36Tyr mutation interfering with the function of the protein is very low, unlike p.Cys61Gly [21] which impairs the interaction of BRCA1 with BARD1 [47]. In contrast to the bioinformatics tools, the functional work we have described demonstrates that the p.Ser36Tyr mutation interferes with the function of the BRCA1 protein. We have used our expression system of full length BRCA1 protein in HEK293T cells and S-phase cell cycle synchronization of BRCA1-BARD1 co-transfected cells to classify a novel BRCA1 VUS identified in Cypriot families, following the guidelines for functional assays recently suggested by Millot and colleagues [30]. Our results indicate that the p.Ser36Tyr BRCA1 variant has abrogated function in functional assays because it is produced at lower levels than wild-type protein and hence it exhibits reduced ability to interact with BARD1, because a high proportion of this variant is withheld in the cytosol instead of migrating to the nucleus following genotoxic stress. In addition, this variant does not promote the formation of conjugated ubiquitin foci at sites of damaged DNA nor it co-localizes with conjugated ubiquitin foci. Considering all data available for the p.Ser36Tyr BRCA1 variant (*in silico* predictions, clinical data, and functional assay results), it should be classified as a moderate cancer risk mutation.

We believe that the described system is reliable for assessing the functional significance of any specific VUS and it can be easily adapted for the classification of VUS identified in the different domains of the *BRCA1* gene. Its advantage is that high protein levels of full-length BRCA1 can be achieved in a transient expression system, thus avoiding long periods of selecting positive clones as described by Chang and colleagues in their mouse embryonic stem cell system [42]. The use of a bicistronic expression vector further facilitates analysis, as through the incorporation of a gene that encodes a fluorescent protein in one of the multiple cloning sites, transfection efficiency can be calculated and immunofluorescence analysis can be restricted to transfected cells only. This system is versatile as it allows the simultaneous evaluation of many of the known BRCA1 key cellular functions. These include screening for protein expression levels, interaction with BARD1, sub-cellular localization and ability to induce the formation of conjugated ubiquitin chains at sites of DNA damage. This system is potentially very powerful as a wide range of information can be generated from just two independent experiments; immunoprecipitation and immunofluorescence staining of transfected cells. For example, using a repertoire of appropriate antibodies against other BRCA1 interacting proteins, the ability of BRCA1 to form the BRCC complex (BRCA2, PALB2, Rad51 and BRCC36) or interact with Abraxas, BACH1 and CtIP at specific stages of the cell cycle can also be investigated. From an immunofluorescence experiment of transiently transfected cells, information such as sub-cellular localization and co-localization with other proteins involved in the DNA repair machinery can be extracted.

In addition, this system can be easily adapted for other assays and for different variants. The VUS can be created by site directed mutagenesis of the wild type encoding construct and for the DNA repair of DSBs by homologous recombination or transcriptional activation assays, certain features of the constructs including the promoter can be incorporated into the appropriate vectors thus examining full-length BRCA1 variant. Furthermore drug sensitivity assays can be performed after modifying the constructs. For example the fluorescent protein marker can be removed from the vector and replaced with an antibiotic resistance marker. This would facilitate the selection of positive clones and the generation of stable transfectants required for drug sensitivity assays. Examining known pathogenic variants in regions other than the BRCA1 RING domain using the above described system will establish this system as a robust method for the clinical evaluation of any BRCA1 VUS. It is our aim to further pursue this and examine a number of different VUS detected in the different functional domains of the BRCA1 protein.

Accurate classification of BRCA1 VUS is important for appropriate genetic counseling and further management of mutation carriers. We have demonstrated via several functional assays that the BRCA1 p.Ser36Tyr variant abrogates BRCA1 protein function. However, the *in silico*, clinical, genetic and epidemiological data which accompany this variant are inconsistent with features of a high-risk pathogenic mutation. Spurdle *et al* [62] were the first to report a BRCA1 variant which is associated with breast/ovarian cancer risk at significantly lower levels compared to truncating BRCA1 mutations. Therefore, they have introduced the term “moderate risk variant” to describe its clinical impact. Taken together, the currently available data indicate that the BRCA1 p.Ser36Tyr variant could be considered as a variant that confers a moderate risk for breast/ovarian cancer. Our findings have important implications for the subsequent counseling and management of p.Ser36Tyr carriers, since they have an increased risk over the general population for developing breast/ovarian cancer, although this risk is lower than that observed for pathogenic BRCA1 mutations. The current estimated risk is OR 3.5, but with lower confidence limit 1.1, indicating that additional genetic studies are necessary to refine risk associated with this variant. Further studies are needed in order to establish guidelines for the clinical management of patients who carry moderate risk BRCA variants and in this context, as recently suggested for another variant, the BRCA1 p.Arg1699Gln, counseling for p.Ser36Tyr could be similar to that for other moderate-penetrance genes such as *CHEK2* and *PALB2* [62].

In conclusion, we provide evidence that the BRCA1 p.Ser36Tyr variant abrogates BRCA1 protein function and based on clinical, epidemiological, genetic and functional assay data we suggest that this variant is associated with a moderate risk of breast and ovarian cancer.

## Supporting Information

Figure S1 **BARD1 expression levels following co-transfection with BRCA1 in S-phase synchronized cells.** Bar charts comparing the protein expression levels of BARD1 exhibited in cells co-transfected with the variants S36Y and C61G or empty vector (EV) against those co-transfected with wild type BRCA1. Samples were normalized with β-actin control. The results are representative of 3 experiments.
(TIF)

Figure S2 **Immunoblot analysis demonstrating the purity of nuclear (NF) and cytosolic (CF) extracts.** The membranes were blocked and stained with antibodies against GAPDH, which is mainly expressed in the cytoplasm and against the nuclear envelope proteins lamin A/C. Staining demonstrated that the nuclear extracts were very pure, as the levels of GAPDH detected were quite low as expected and the cytoplasmic extracts were not contaminated with nuclear proteins as lamin A/C was not detected. The results are representative of 2 experiments.
(TIF)

Figure S3 **p.Cys61Gly (C61G) BRCA1 variant exhibits residual activity.** Immunofluorescence analysis of transfected, S-phase synchronized cells demonstrated that the p.Cys61Gly BRCA1 variant fails to co-localize with conjugated ubiquitin detected by the FK2 antibody that recognizes only conjugated ubiquitin structures. A high proportion of p.Cys61Gly BRCA1 and conjugated ubiquitin were detected outside the nucleus similar to wild type transfected cells (Figure 5). HU treatment induced mobilization of the p.Cys61Gly variant in the nuclei of transfected cells but not of conjugated ubiquitin. A high proportion of conjugated ubiquitin remained perinuclear, although in some cells co-localization with p.Cys61Gly in the nuclei was observed, indicating some residual activity. Empty vector (EV) control demonstrated that in the absence of BRCA1 the levels of conjugated ubiquitin foci formed in the nuclei were decreased compared to BRCA1 transfected cells. Nuclei were stained with DAPI. The forth column is the merge of BRCA1 and conjugated ubiquitin and where green and red signals overlap a yellow signal is seen indicating co-localization. The fifth column is the merge of all stains. Where all signals overlap a white signal is seen, where red and blue signal overlap a pink signal is seen and where green and blue signals overlap a violet signal is seen indicating co-localization. Statistical analysis confirmed that the observed effects are significant (p<0.05). Insets show the arrow pointed cells following enlargement. The results are representative of 3 experiments. Scale bar: 40 μm.
(TIF)

Supporting Information S1 **A detailed description of Plasmid Construct design, Western Blot and Co-precipitation analysis.**
(DOC)
