# Supplementary figures and images for: The BRCA1 Variant p.Ser36Tyr Abrogates BRCA1 Protein Function and Potentially Confers a Moderate Risk of Breast Cancer
Source: PLoS One. 2014 Apr 2;9(4):e93400. doi: 10.1371/journal.pone.0093400 (PMC3973689; doi:10.1371/journal.pone.0093400)

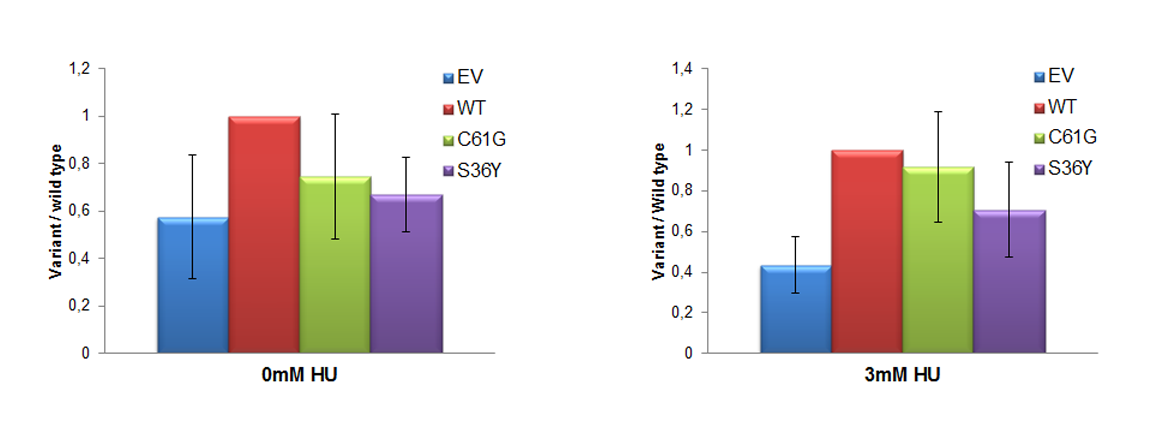

Supplement: Figure S1 — BARD1 expression levels following co-transfection with BRCA1 in S-phase synchronized cells. Bar charts comparing the protein expression levels of BARD1 exhibited in cells co-transfected with the variants S36Y and C61G or empty vector (EV) against those co-transfected with wild type BRCA1. Samples were normalized with β-actin control. The results are representative of 3 experiments. (TIF) [file pone.0093400.s001.tif]

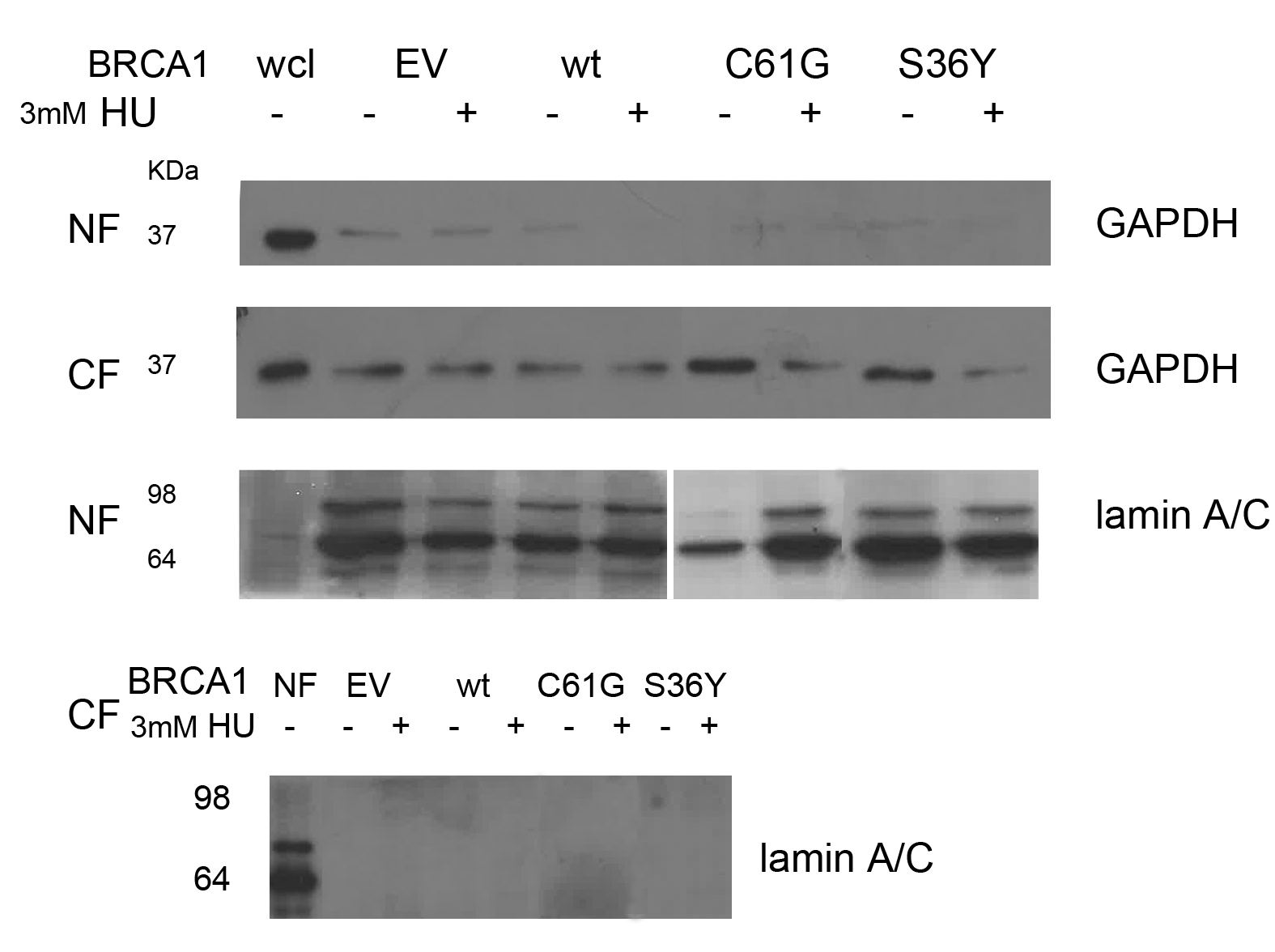

Supplement: Figure S2 — Immunoblot analysis demonstrating the purity of nuclear (NF) and cytosolic (CF) extracts. The membranes were blocked and stained with antibodies against GAPDH, which is mainly expressed in the cytoplasm and against the nuclear envelope proteins lamin A/C. Staining demonstrated that the nuclear extracts were very pure, as the levels of GAPDH detected were quite low as expected and the cytoplasmic extracts were not contaminated with nuclear proteins as lamin A/C was not detected. The results are representative of 2 experiments. (TIF) [file pone.0093400.s002.tif]

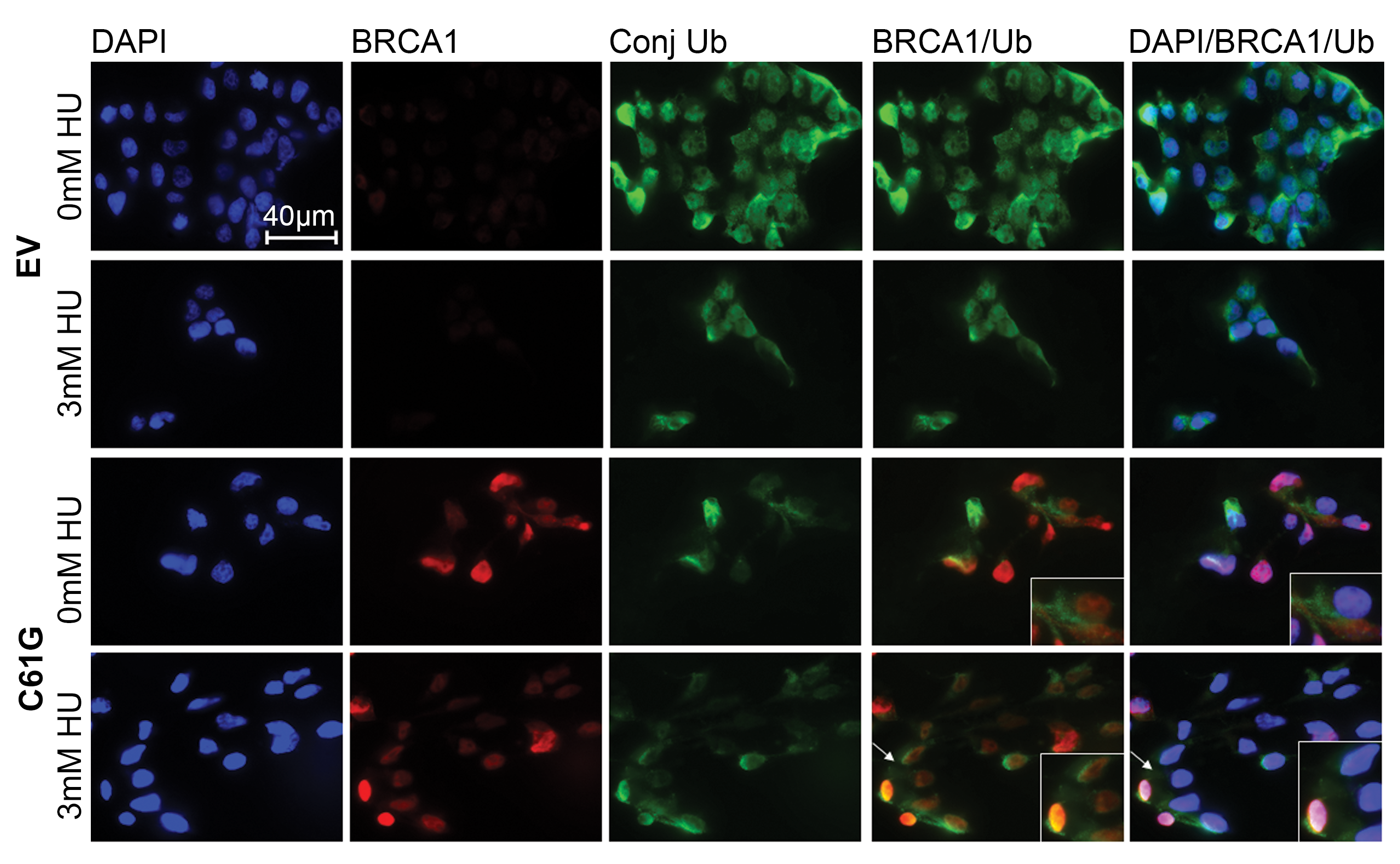

Supplement: Figure S3 — p.Cys61Gly (C61G) BRCA1 variant exhibits residual activity. Immunofluorescence analysis of transfected, S-phase synchronized cells demonstrated that the p.Cys61Gly BRCA1 variant fails to co-localize with conjugated ubiquitin detected by the FK2 antibody that recognizes only conjugated ubiquitin structures. A high proportion of p.Cys61Gly BRCA1 and conjugated ubiquitin were detected outside the nucleus similar to wild type transfected cells (Figure 5). HU treatment induced mobilization of the p.Cys61Gly variant in the nuclei of transfected cells but not of conjugated ubiquitin. A high proportion of conjugated ubiquitin remained perinuclear, although in some cells co-localization with p.Cys61Gly in the nuclei was observed, indicating some residual activity. Empty vector (EV) control demonstrated that in the absence of BRCA1 the levels of conjugated ubiquitin foci formed in the nuclei were decreased compared to BRCA1 transfected cells. Nuclei were stained with DAPI. The forth column is the merge of BRCA1 and conjugated ubiquitin and where green and red signals overlap a yellow signal is seen indicating co-localization. The fifth column is the merge of all stains. Where all signals overlap a white signal is seen, where red and blue signal overlap a pink signal is seen and where green and blue signals overlap a violet signal is seen indicating co-localization. Statistical analysis confirmed that the observed effects are significant (p<0.05). Insets show the arrow pointed cells following enlargement. The results are representative of 3 experiments. Scale bar: 40 μm. (TIF) [file pone.0093400.s003.tif]
