# Supplementary material for: The BRCA1 Variant p.Ser36Tyr Abrogates BRCA1 Protein Function and Potentially Confers a Moderate Risk of Breast Cancer
Source: PLoS One. 2014 Apr 2;9(4):e93400. doi: 10.1371/journal.pone.0093400 (PMC3973689; doi:10.1371/journal.pone.0093400)
Supplement: Supporting Information S1 — A detailed description of Plasmid Construct design, Western Blot and Co-precipitation analysis. (DOC) [file pone.0093400.s004.doc]

**Supporting Information S1**

**Title**

The BRCA1 variant p.Ser36Tyr abrogates BRCA1 protein function and potentially confers moderate risk of breast cancer

**Authors**

Charita M. Christou, ^1†^, Andreas Hadjisavvas, ^1†^, Maria Kyratzi, ^1,2^, Christina Flouri, ^1^, Ioanna Neophytou, ^1^, Violetta Anastasiadou, ^3^, Maria A. Loizidou, ^1^, and Kyriacos Kyriacou,^1*^

**1** The Cyprus Institute of Neurology and Genetics, Department of Electron Microscopy / Molecular Pathology, Nicosia, Cyprus.

**2** The University of Cyprus, Department of Biological Sciences, Nicosia, Cyprus.

**3** The Cyprus Institute of Neurology and Genetics, Department of Clinical Genetics, Nicosia, Cyprus.

**†** These authors equally contributed to the work.

**^*^Correspondence to:** Kyriacos Kyriacou, E-mail: [kyriacos@cing.ac.cy](mailto:kyriacos@cing.ac.cy), Tel: 0035722392631, Fax: 0035722392641

**Materials and Methods**

Plasmid construction

The vector pCDNA3.1 encoding full length BRCA1 cDNA sequence (kind gift from Dr Wu, UC, Irvine) was checked and corrected. The corrected vector was used as a template for all plasmid construction. Full-length BRCA1 (Accession number NM_007294.3) was initially sub-cloned into the pFLAG-CMV2 vector (kind gift from Dr Santama, UCY, Cyprus) in a two step cloning process. In the first step the DNA fragment containing residues 1 to 2317 was amplified by PCR and cloned into the pFLAG-CMV2 vector between *NotI* and *KpnI* restriction sites. The PCR was carried out in a final volume of 50 μl using 50 ng template DNA, 0.25 mM dNTPs, 1 x *Pfu* buffer, 2 units *Pfu* polymerase (Fermentas UAB, ThermoScientific, Vilnius, Lithuania), 2.5 μl DMSO and 10 pmol of the following forward and reverse primers:

5’-AAGGAAAAAA*GCGGCCGC*GATGGATTTATCTGCTCTTCGCG-3’ and

5’- TACGG*GGTACC*AATGAAATACTGCTACT-3’.

DNA was amplified in a Veriti thermal cycler (Applied Biosystems, Life Technologies, Foster City, USA) using the following protocol: 95^o^C for 5 min; 95^o^C, 30 sec; 55^o^C 30 sec; 72^o^C 4.5 min for 30 cycles, followed by a final extension step at 72^o^C for 10 min. The amplified product was digested with *NotI* and *KpnI* (New England Biolabs, Massachusetts, USA) and cloned into pFLAG-CMV2 digested with the same enzymes. The resulting plasmid was designated as pCC031. In the next step the BRCA1 fragment from 2317 to 5592 (end) was PCR amplified as above using the following forward and reverse primers:

5’-TACGGGGTACCTGGTACTGATTATGG-3’ and

5’-TATCGC*GGATCC*TCAGTAGTGGCTGTGGGGGA-3’.

It was then digested with *KpnI* and *BamHI* and sub-cloned into pCC031 digested with the same enzymes, thus resulting in a vector encoding full-length N-terminally Flag-tagged BRCA1, designated as pCC032. To facilitate cloning, the *NotI* site of the construct was ablated and created a *NaeI* site by performing PRC-based site-directed mutagenesis. The PCR was carried out in a final volume of 50 μl using 50 ng template DNA (pCC032), 0.25 mM dNTPs, 1 x *Pfu* buffer, 2 units *Pfu* polymerase (Fermentas UAB, ThermoScientific, Vilnius, Lithuania), and 15 pmol of the following forward and reverse primers:

5’-GACGACAAGCTTGCGCCGGCGATGGATTTATCTGC-3’ and

5’- GCAGATAAATCCATCGCCGGCGCAAGCTTGTCGTC-3’.

The following PCR parameters were used to incorporate the mutations: 95^o^C 1 min; 95^o^C 30 sec; 55^o^C 1 min; 68^o^C 10.5 min for 16 cycles. Following *DpnI* digestion of the original plasmid DNA, 2 μl of reaction containing the new mutated plasmid DNA were used to transform competent *Escherichia coli* DH5α cells. Plasmid DNA was extracted using the Qiagen Plasmid Mini Prep Kit according to the manufacturer’s instructions (Qiagen, GmbH, Hilden, Germany) and designated as plasmid pCC033.

PCR-based site-directed mutagenesis was employed to introduce the VUS mutation c.107C>A (p.Ser36Tyr) and the c.181T>G (p.Cys61Gly) pathogenic mutation, to act as an experimental control (Brzovic, et al., 1998). The following pairs of forward and reverse primers were used:

5’-CAAGGAACCTGTCTACACAAAGTGTGACC-3’ and

5’- GGTCACACTTTGTGTAGACAGGTTCCTTG-3’ for p.Ser36Gly and

5’-GGGCCTTCACAGGGTCCTTTATGTAAG and

5’- CTTACATAAAGGACCCTGTGAAGGCCC-3’ for p.Cys61Gly.

The resulting constructs were designated as pCC037 and pCC047 for p.Ser36Gly and p.Cys61Gly mutations respectively.

In order to verify successful transfection, N’-Flagged BRCA1 was sub-cloned into the MCSI of the retroviral vector pQCXIX between *NotI* and *AgeI* restriction sites. The vector carries the dsRED gene in MCSII (kind gift of Prof. O’Callaghan, University of Oxford). The primers used to PCR amplify N-flagged BRCA1 from the plasmid templates pCC035 and pCC037 are: 5’-AAGGAAAAAA*GCGGCCGC*ATGGACTACAAAGACGATGACGAC-3’ and 5’-TATAAT*ACCGGT*TCAGTAGTGGCTGTGGGGGA-3’. The resulting plasmids were designated as pCC035 (wt BRCA1 in pQCXIX) and pCC045 (p.Ser36Gly BRCA1 UV in pQCXIX).

Full-length BARD1 cDNA was sub-cloned into the MCSI of pQCXIX vector between *NotI-AgeI* using pCMV6-entry vector containing the ORF of BARD1 (OriGene Technologies Inc, Rockville, USA). The following forward and reverse primers were used to PCR amplify BARD1:

5’-AAGGAAAAAA*GCGGCCGC*ATGCCGGATAATCGGCAGCC-3’ and

5’ATCC*TTAATTAA*GGTGATGGTGATGGTGATGACCGGTGCTGTCAAGAGGAAGCAACTC-3’. The resulting plasmid was designated pCC050.

All PCR primers were obtained from Metabion GmbH, Martinsried, Germany. The sequences of all constructs were confirmed using a 3130xL Genetic Analyzer (Applied Biosystems, Life Technologies, Foster City, USA).

Western Blotting and Co-precipitation analysis

The density of each band was calculated using Tinascan software. Expression levels were normalized by dividing the density of each band with the corresponding density of the β-actin loading control band. For co-precipitation analysis, the density calculated for BARD1 co-precipitated with wild-type BRCA1 at untreated or untreated conditions was considered to be 100%. Densities calculated for BARD1 co-precipitated with p.Ser36Gly and p.Cys61Gly variants were then divided by the corresponding density of BARD1 precipitated with wild-type BARD1 thus obtaining the fold-number of increase or decrease in co-precipitated BARD1 by p.Ser36Gly or p.Cys61Gly variants compared to wild type BRCA1.
